# The Climate Crisis Will Not Wait

**DOI:** 10.1007/s10272-020-0934-9

**Published:** 2020-12-01

**Authors:** Matilda Gettins

**Affiliations:** Fridays For Future München, Seidelbaststr. 12, 80939 München, Germany

## Abstract

Climate communication is an essential step towards changing the rules. Policies with a large-scale impact on the economy and individuals, such as a high carbon tax, must be justified in terms of their proportionality.

COVID-19 has defined the year 2020. The pandemic has dominated headlines, created an economic shutdown and became political priority number one. Due to the intense focus on the pandemic, there has been less bandwith left over for the greatest existential threat of our time: the climate crisis.

Despite the best attempts to argue that the coronavirus crisis has been advantageous for addressing climate issues, this is not the case. As media coverage shifted from the climate crisis to the pandemic, public scrutiny waned and governments reduced their already minimal efforts at climate policy. Economic stimulus packages were drafted and implemented – with more money flowing into fossil fuels than into renewable energies. The economic downturn, though reducing emissions temporarily, had strong adverse effects. Firms and investors are not keen to invest in times of crisis and those with plans to increase their sustainability were prevented by financial constraints. And the public, consumed with health, employment and educational worries among others, had little attention span for yet another disaster.

## The coronavirus taught us the urgency of a crisis

The COVID-19 pandemic had disastrous effects around the entire world. However, the relative success or failure of various governments in their pandemic responses illustrates the crucial importance of taking early action in a crisis. On average, those countries that implemented preventative measures by reducing the risk of rapid exponential growth of COVID-19 infections in order to spare their health care systems from collapse fared better in terms of mortality as well as economic impact.

### Tipping points of no return

This bears a strong resemblance to the climate crisis. Our climate system contains similar tipping points that, once triggered, are irreversible and worsen the effect of the climate crisis beyond the control of human impact. The melting of ice sheets in Greenland, causing a reduction of the albedo effect and hence increasing the rate of melting, is but one of many examples (Umweltbundesamt, 2008). To ensure that we do not reach the exponential domino effect caused by these tipping points – which would, amongst many disastrous consequences, increase the geographic range of malaria and other vector-borne diseases – we need to avoid reaching more than 1.5 degrees Celsius warming at all costs (IPCC, 2018). To do this, it is necessary to act now.

We need to start by changing the rules. Many firms and countries continue to emit greenhouse gases at little or no cost. This market failure is disastrous in its consequences and should have been balanced out through public intervention long ago. In fact, the effects of not solving the greenhouse gas externality are detrimental in two additional aspects.

Firstly, firms that emit greenhouse gases lack economic incentives to transition to clean energies while remaining profitable. If they do transition, it is usually out of corporate or executive values or public pressure. However, they find that this often leads to an economic disadvantage, essentially punishing them for making the responsible decision. In addition, those firms that cannot afford these additional costs are wholly unable to transition to clean energies.

Secondly, the same theory applies to individuals. If there are no additional costs connected with to flying or to eating meat, there is little incentive to resist temptation and change behaviour. The prices of goods should reflect their environmental costs. Furthermore, there should be environmentally friendly alternatives that are also affordable to the general public so that anyone who would like to live in a more climate-compatible manner, can afford to do so and live in line with their values.

### Crisis communication

Climate communication is an essential step towards changing the rules. Policies with a large-scale impact on the economy and individuals, such as a high carbon tax, must be justified in terms of their proportionality. Regarding the severity of the climate crisis, the scientific community is effectively in agreement that this would be a proportional measure.[1] However, it is the role of government not only to draft and implement measures to mitigate the crisis, but also to communicate them.

During the coronavirus pandemic, this crisis communication was done a lot better than it has been in climate politics for the last decades. Politicians used live address to reach the

© The Author(s) 2020. Open Access: This article is distributed under the terms of the Creative Commons Attribution 4.0 International License (https://creativecommons.org/licenses/by/4.0/).

Open Access funding provided by ZBW – Leibniz Information Centre for Economics.

**Matilda Gettins**, Fridays for Future Munich, Germany.

1 See for example Carbon Tax Center (2015).

public, created expert panels and provided educational content for children. This process of communication was supported by the media who constantly updated the public and provided the necessary background information based on the latest scientific research and reporting. As a result, there was a general understanding of the degree of vulnerability to the crisis and an acceptance of drastic interventions, such as national lockdowns.

### Fighting the climate crisis

Just as with surmounting the COVID-19 crisis, fighting the climate crisis is an immense task.

The EU needs to reform agriculture to net-zero carbon emissions. Energy sources must be made entirely renewable. We have to implement a sustainable transport revolution. The list goes on and on.

While these goals may be "ambitious", they are nonetheless necessary. And they are possible. The Wuppertal Institute (2020) shows, for example, the transition pathway Germany could take to fulfill its commitment to the Paris Agreement.

However, just because a green transition is possible does not mean that it will happen. In order to avoid a climate emergency, we must not give up. Instead, when drafting policies for the pathway to recovery after COVID-19, we need to include measures to fight the climate crisis as well. We need to use synergic effects such as investments in mobility or renewable energies.

While there is no denying that COVID-19 has defined 2020, we cannot let it lead us directly into the heart of an inevitable disaster: we must tackle the climate crisis head on.
